# Novel autoimmune response in a tauopathy mouse model

**DOI:** 10.3389/fnins.2013.00277

**Published:** 2014-01-10

**Authors:** Carlos J. Nogueras-Ortiz, Hector J. De Jesús-Cortes, Jaime Vaquer-Alicea, Irving E. Vega

**Affiliations:** ^1^Department of Biology, University of Puerto Rico – Río Piedras CampusSan Juan, PR, USA; ^2^Department of Chemistry, University of Puerto Rico – Río Piedras CampusSan Juan, PR, USA

**Keywords:** Alzheimer's disease, tauopathies, tau-mediated neurodegeneration, autoimmune response, biomarkers, diagnostics

## Abstract

Molecular diagnostic tools with non-invasive properties that allow detection of pathological events in Alzheimer's disease (AD) and other neurodegenerative tauopathies are essential for the development of therapeutics. Several diagnostic strategies based on the identification of biomarkers have been proposed. However, its specificity among neurodegenerative disorders is disputable as the association with pathological events remains elusive. Recently, we showed that Amphiphysin-1 (AMPH1) protein's abundance is reduced in the central nervous system (CNS) of the tauopathy mouse model JNPL3 and AD brains. AMPH1 is a synaptic protein that plays an important role in clathrin-mediated endocytosis and associates with BIN1, one of the most important risk loci for AD. Also, it has been associated with a rare neurological disease known as Stiff-Person Syndrome (SPS). Auto-antibodies against AMPH1 are used as diagnostic biomarkers for a paraneoplastic variant of SPS. Therefore, we set up to evaluate the presence and abundance of auto-AMPH1 antibodies in tau-mediated neurodegeneration. Immunoblots and enzyme-linked immunosorbent assays (ELISA) were conducted to detect the presence of auto-AMPH1 antibodies in sera from euthanized mice that developed neurodegeneration (JNPL3) and healthy control mice (NTg). Results showed increased levels of auto-AMPH1 antibodies in JNPL3 sera compared to NTg controls. The abundance of auto-AMPH1 antibodies correlated with motor impairment and AMPH1 protein level decrease in the CNS. The results suggest that auto-AMPH1 antibodies could serve as a biomarker for the progression of tau-mediated neurodegeneration in JNPL3 mice.

## Introduction

Tauopathies are a group of neurodegenerative disorders that present cognitive and/or motor deficits associated to modifications and oligomerization of the microtubule-associated protein tau. Alzheimer's disease (AD) is the most common and intensively studied tauopathy because of its prevalence, severity, and care costs (Steenland et al., 2009). These facts instigate special attention to AD's etiology and associated molecular mechanisms to identify potential diagnostic and therapeutic strategies. At present time, AD diagnosis is possible at mid to late stages of progression when symptoms can be clearly identified by physicians using the available psychometric tests. However, approximately 10% of the cases can be unrecognized by most experienced physicians and related neurodegenerative disorders could be misidentified as AD (Beach et al., 2012). Other diagnostic strategies consist of neuroimaging techniques with high costs and yet unproven accuracy (McKhann et al., 2011). Additional diagnostic tools are based on the detection of disease biomarkers in cerebrospinal fluid (CSF) collected by lumbar puncture, a painful and invasive procedure that can cause serious health complications (Blennow et al., 2012). Therefore, it is imperative to identify accurate biomarkers of tau-mediated neurodegeneration with non-invasive properties in order to develop definitive diagnostic tools for AD and related disorders.

Previously, we reported that the abundance of the synaptic protein Amphiphysin-1 (AMPH1) is reduced in the central nervous system (CNS) of the JNPL3 tauopathy mouse model and AD brains (De Jesus-Cortes et al., 2012). The putative pathological significance of AMPH1 protein level reduction relies on the key role it plays in clathrin-mediated synaptic vesicle endocytosis, an essential event for synaptic function (Wu et al., 2009). Consistently, *AMPH1* knockout (KO) mice shows decreased synaptic vesicle recycling and cognitive impairment which is a clinical feature in AD (Di Paolo et al., 2002). Deletion of *AMPH1* is accompanied by decreased expression of AMPH2, also known as BIN1. Interestingly, single nucleotide polymorphism at *BIN1* has recently been identified as one of the most important risk factors for late-onset AD (Bertram et al., 2007). These observations suggest a link between AMPH1 and tau-mediated neurodegeneration.

In addition to the association with neurodegeneration in JNPL3 mice and AD, AMPH1 has been implicated in another neurological disorder known as Stiff-Person Syndrome (SPS) (Murinson and Guarnaccia, 2008; Geis et al., 2009, 2010). SPS is classified as an autoimmune disease characterized by severe spasms and thoracolumbar stiffness due to hypersensitivity to stimuli such as noise, touch, and emotional distress. SPS patients are divided in two groups: those with anti-glumatic acid decarboxylase (GAD) antibodies and others with anti-AMPH1 antibodies in serum. SPS patients with anti-AMPH1 antibodies are symptomatically different from anti-GAD positive patients in that they are older and show severe cervical stiffness. Although the pathophysiological process that leads to the generation of anti-AMPH1 positive SPS is still unknown, we hypothesize that AMPH1 depletion in tauopathy could lead to the secretion or release of endogenous AMPH1 peptides triggering an autoimmune response. This hypothesis laid the foundation to evaluate the association between auto-AMPH1 antibodies and AMPH1 protein level depletion on the course of neurodegeneration in the JNPL3 tauopathy mouse model.

## Materials and methods

### Animal model and assessment of motor impairment

The JNPL3 mouse model expresses the human tau isoform 0N4R bearing the P301L mutation (hTauP301L), a pathological molecule in human tauopathies. hTauP301L is commonly found in post-mortem human brain tissue from patients with frontotemporal dementia and parkinsonism linked to chromosome 17 (Lewis et al., 2000). JNPL3 mice develop tau pathology in an age-dependent and brain region-specific manner (Lewis et al., 2000). The JNPL3 and non-transgenic (NTg) control mice used in this study were euthanized at the age reported on Table S1 (supplementary material).

Lewis and McGowan have designed a scale, ranging from 0 to 30, that rates motor dysfunction in JNPL3 mice based on their performance on tail hang, rope hang, and righting reflex tests (Lewis and McGowan, 2005). The Lewis and McGowan's scale was used to evaluate the motor abilities of JNPL3 hemizygous and NTg littermates ranging from 2.9 to 13 months of age (Table S1 supplementary material). Mice with motor impairment score equal to or below 12 were catalogued as normal, whereas mice with score 13 or above where catalogued as motor impaired. For this study, the experiments were performed using different cohorts based on age and motor impairment score, irrespectively of their genotype. This approach allowed us to conduct the same experiments in cohorts of mice with similar age and motor impairment score, which contributes to internally validate the results obtained. It is important to mention that mice in a cohort were discarded only if the result obtained was identified as an outlier within the specific cohort used in an experiment. The University of Puerto Rico animal care and use committee approved the protocol (403-2006) depicting the experiments done in this study.

### Sera extraction and spinal cord protein lysate preparation

Blood and spinal cords were extracted from euthanized mice. Blood was allowed to coagulate for 1 h at room temperature. Afterwards, the coagulated blood was centrifuged at low speed (2300 × g) for 5 min and supernatants stored in aliquots at −20°C. Spinal cord protein lysates were prepared by grinding the tissue in Buffer A (20 mM Tris base, pH 7.4, 150 mM NaCl, 1 mM EDTA, 1 mM EGTA, 1 mM phenylmethylsulfonyl fluoride, 5 mM sodium pyrophosphate, 30 mM β-glycerol phosphate, and 30 mM sodium fluoride; volume: five times the wet weight), followed by centrifugation at 14,000 rpm (21,000 × g) for 10 min. The supernatant was transferred to a clean tube and used for further analyses.

### Immunoblots

Immunoblots were conducted as described by De Jesus-Cortes et al. (2012). Anti-AMPH1 (1:1000; Novus Biologicals, cat. no. NB110-55455) and anti-GAPDH (1:1000; Abcam, cat. no. 9484) antibodies were used for densitometric analyses. Tau13 antibody recognizing human tau (1:50000; Covance, cat. no. MMS-520R-500) was used to confirm mice genotyping and to assess the accumulation of pathological tau species in the spinal cords. Secondary antibodies: HRP-conjugated anti-mouse (Millipore, cat. no. AP181P) and anti-rabbit (Millipore, cat. no. MAB201P) antibodies.

### Indirect ELISA

Sera were subjected to indirect enzyme-linked immunosorbent assay (ELISA), according to Wilkin et al. (1985). Briefly, the wells of 96-well microplates (for Figure 3A was used BD Biosciences, cat. no. 353228; for Figure 3D was used Fisher Scientific, cat. no. 12565501) were coated with 300 ng of recombinant AMPH1 (Novus Biologicals, cat. no. H00000273-P01) overnight at 4°C. Coating efficiency experiments indicated that 300 ng of the antigen per well are sufficient for surface saturation (data not shown). Non-specific binding was blocked using 3% bovine serum albumin in PBS-1X supplemented with 0.05% Tween-20 for 1h at room temperature. Afterwards, a 1:100 dilution in blocking solution of each serum was added to each well containing bound recombinant AMPH1. Anti-mouse IgG antibodies conjugated to horseradish peroxidase (Millipore, cat. no. 12-349) and colorimetric HRP substrate (Bio-Rad, cat. no. 172-1064) were used for the detection of bound auto-AMPH1 antibodies. The background signal was different in the two types of microplates used. Therefore, the absorbance detected in the experimental samples was corrected using the background signal (no antigen) and normalized to input (recombinant AMPH1 recognized by anti-AMPH1 commercial antibody instead of sera) as internal control.

### Statistical analyses

ELISA results were subjected to a ROUT outlier test with *Q* = 0.1% to remove definite outliers. Means were compared by Two-Way ANOVA (Figures 2B, 3A) and paired two-tailed *t*-test (Figure 3D) with a 95% confidence interval. The Pearson's correlation test was used to evaluate the association between independent variables. Two-tailed *P* values with a 95% confidence interval were calculated for every correlation. Statistical analyses were performed using Graphpad Prism 6.

## Results

**Figure 1:**
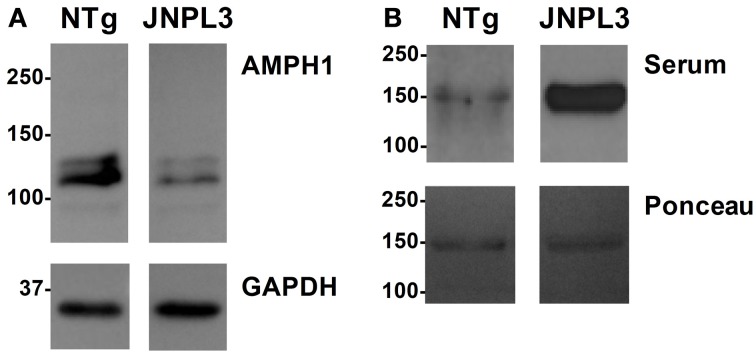
**AMPH1 protein level decrease in the CNS is accompanied by increased levels of auto-AMPH1 antibodies in a JNPL3 mouse**. **(A)** The abundance of AMPH1protein is significantly reduced in the spinal cord of a JNPL3 mouse in comparison to a non-transgenic (NTg) littermate. **(B)** Serum from a JNPL3 mouse with motor impairment and a normal NTg littermate were collected and used to recognize recombinant AMPH1 (Ponceau). Blots show increased levels of auto-AMPH1 antibodies in the serum of the JNPL3 mouse that showed AMPH1 protein level reduction in the spinal cord, but not the serum derived from the NTg mouse.

The immune reaction against proteins or other molecules that are endogenous (i.e., self-produced) to the organism generates auto-antibodies (or self-antibodies). These antibodies are indicative of an actual pathological event thus serving as molecular diagnostic tools. The reduction of AMPH1 protein abundance in JNPL3 mice (De Jesus-Cortes et al., 2012) suggests a putative role in the pathobiology of tau-mediated neurodegeneration. Based on the fact that auto-AMPH1 antibodies are used as diagnostic tool in SPS, we set to study the presence of auto-AMPH1 antibodies in the serum of JNPL3 mice, using recombinant AMPH1 as “bait.” Serum obtained from a 12 month old terminally ill JNPL3 mouse with reduced abundance of AMPH1 protein in the spinal cord (Figure 1A) was used for immunoblot assays; serum from a NTg littermate with normal protein levels of AMPH1 was used as control (Figure 1A). The results showed that auto-AMPH1 antibodies are readily detected in serum from JNPL3 mice with reduced abundance of AMPH1 protein, but not in NTg controls (Figure 1B).

We proceed to evaluate the association between AMPH1 protein level changes in the CNS and the emergence of auto-AMPH1 antibodies. Lewis et al. (2000) demonstrated that motor impairment progresses with age and correlates with neurodegeneration in the spinal cords of JNPL3 mice. Motor impairment also correlates with the abundance of tau aggregates known as neurofibrillary tangles (NFTs) which are considered to be the pathological hallmark in AD and related disorders. Thus, motor impairment in JNPL3 mice is a reliable marker for the progression of tau-mediated neurodegeneration. Impaired motor behavior includes slowed or impaired righting when placed supine, dystonia of tail and hind limbs, abnormal extension of hind legs following tail elevation, and hunched posture. A motor impairment scale (see Materials and Methods) was used a behavioral marker to assess the amount of AMPH1 in the spinal cord and auto-AMPH1 antibodies as the severity of neurodegeneration progressed.

**Figure 2:**
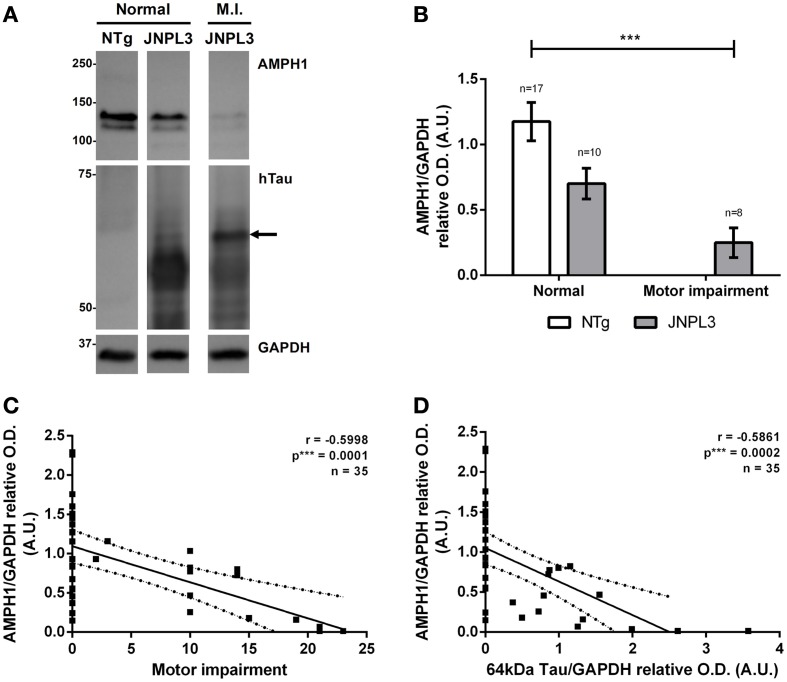
**AMPH1 protein depletion is associated with pathological events in tau-mediated neurodegeneration. (A)** When compared to NTg controls, JNPL3 mice with impaired motor function show reduced levels of the protein AMPH1 in the spinal cord, as illustrated by immunoblots. The protein level reduction is accompanied by the accumulation of 64kDa hyperphosphorylated hTauP301L. Arrow indicates tau positive bands with a 63.2 kDa molecular weight according to linear semi-logarithmic interpolations. GAPDH chemiluminescence was used as loading control. **(B)** Densitometry analysis of **(A)**. Bar graph shows the mean ± s.e.m. for each group. Means were compared by two-way ANOVA (p^***^ = 0.0007). **(C,D)** AMPH1 protein levels in the spinal cord negatively correlate with motor impairment **(C)** and the accumulation of 64kDa hTauP301L **(D)**. The linear regression of the best fit line shows the 95% confidence band; *R*^2^ = 0.3597 for **(C)** and *R*^2^ = 0.3435 for **(D)**.

As expected, AMPH1 protein level reduction is associated with the progression of neurodegeneration in the mouse model JNPL3 (Figure 2). The results showed that motor impaired JNPL3 mice have reduced levels of AMPH1 in comparison to NTg mice (Figures 2A,B). The apparent negative correlation between AMPH1 protein levels and motor impairment was confirmed statistically (Figure 2C). AMPH1 protein level decrease also correlates with the accumulation of 64 kDa hyperphosphorylated hTauP301L (Figures 2A,D), a positive indicator of tau pathology in JNPL3 mice that concurs with motor dysfunction (data not shown) and neurodegeneration (Lewis et al., 2000; Sahara et al., 2002). These observations confirm the association between AMPH1 protein level reduction and tau-mediated neurodegeneration in the JNPL3 mouse model.

**Figure 3:**
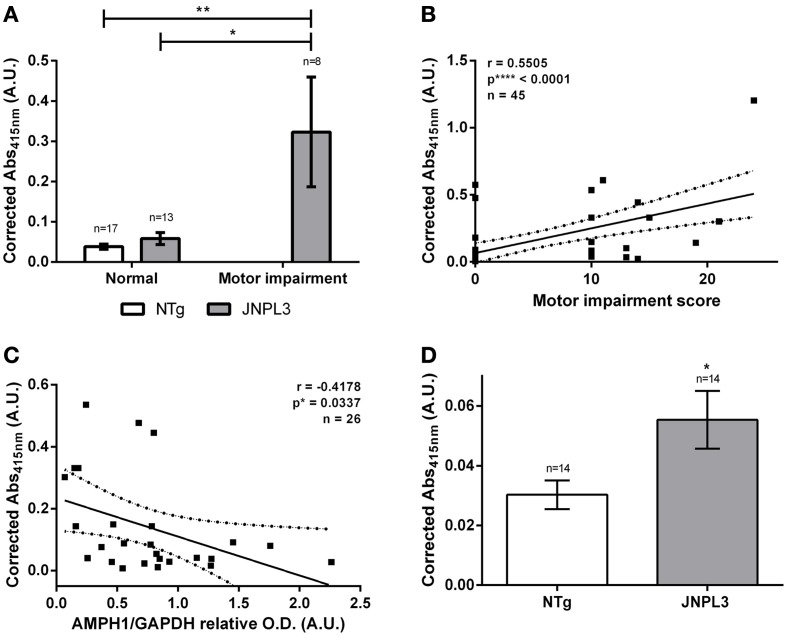
**Auto-AMPH1 antibodies are biomarkers of tau-mediated neurodegeneration in the JNPL3 tauopathy mouse model. (A)** ELISA shows increased levels of auto-AMPH1 antibodies in JNPL3 mice with motor impairment when compared to normal JNPL3 mice and NTg littermates. Bar graph shows the mean ± standard error of the mean (s.e.m.) for each group. Means were compared by two-way ANOVA; p^**^ = 0.0032, p^*^ = 0.0102. **(B,C)** The amount of auto-AMPH1 antibodies in serum positively correlates with motor decline **(B)** and AMPH1 protein depletion in the CNS **(C)**, as indicated by the Pearson's “*r*” correlation analysis. The linear regression of the best fit line shows the 95% confidence band; *R*^2^ = 0.3031 for **(B)** and *R*^2^ = 0.1745 for **(C)**. **(D)** ELISA shows increased levels of auto-AMPH1 antibodies in old JNPL3 mice (8.1–13 months of age) when compared to NTg littermates. Bar graph shows the mean ± standard error of the mean (s.e.m.) for each group. Means were compared by paired two-tailed *t*-test; p^*^ = 0.0378.

Next, we assessed the amount of auto-AMPH1 antibodies as tauopathy progressed, and its association with motor impairment and AMPH1 protein level depletion. Interestingly, the amount of AMPH1 increased with the level of neurodegeneration in JNPL3 mice. JNPL3 mice with motor impairment have higher levels of auto-AMPH1 antibodies than normal JNPL3 and NTg mice (Figure 3A). A positive correlation exists between the level of auto-AMPH1 antibodies and the severity of motor impairment (Figure 3B), indicating that as neurodegeneration progresses more auto-AMPH1 antibodies are detected in serum. Additionally, the abundance of auto-AMPH1 antibodies was found to negatively correlate with AMPH1 protein levels in the spinal cord (Figure 3C), thus providing a link between a neuropathobiological event and an autoimmune response in tau-mediated neurodegeneration. Finally, we quantified anti-AMPH1 antibodies in sera from old JNPL3 mice and NTg littermates to assess auto-AMPH1 antibodies as a biomarker of aging. Results indicate that the signal of auto-AMPH1 antibodies derived from old and motor impaired JNPL3 mice is significantly higher compared to those found in the sera of NTg age-matched littermates (Figure 3D). Hence, the emergence of auto-AMPH1 antibodies is a molecular event associated to tau-mediated neurodegeneration in JNPL3 mice and not to aging.

## Discussion

The adaptive immune system is thought to be a rich source of biomarkers as it reacts to disease-specific antigens through the amplification of antibodies that could be detected in blood samples using relatively non-invasive procedures (Nagele et al., 2011; Reddy et al., 2011). However, the detection of auto-antibodies remains controversial for many diseases, including AD. This is, in part, because the association between the vast majority of these autoimmune responses and pathobiological processes in the brain is still unknown, thus questioning their potential as disease-specific biomarkers. In this regard, we showed the identification of a novel autoimmune response in association with a pathobiological event in the CNS of a tauopathy mouse model.

The featured autoimmune response consists of the overproduction of auto-AMPH1 antibodies in motor impaired JNPL3 mice, compared to normal mice and NTg controls (Figures 1B, 3A,D). The abundance of auto-AMPH1 antibodies statistically correlated with deficiencies in the mice's motor behavior (Figure 3B), a phenotype previously demonstrated to be related to neuronal loss in the spinal cord (Lewis et al., 2000). These results identify auto-AMPH1 antibodies as biomarkers of tau-mediated neurodegeneration in JNPL3 mice. Furthermore, the abundance of auto-AMPH1 antibodies in blood is associated with protein level reduction of AMPH1 in the CNS (Figure 3C), a molecular event linked to pathological events in tauopathy (Figure 2).

Based on our understanding of how autoimmune responses generally occur, we can infer that AMPH1 depletion in tauopathy may trigger a response from the immune system leading to the generation of auto-AMPH1 antibodies that can be detected in blood. Calpain proteases cleave AMPH1 at specific sites under neurotoxic conditions and are known to be hyperactive in AD brains (Camins et al., 2006; Wu et al., 2007). It is plausible to suggest that calpain-mediated cleavage of AMPH1 could lead to the release of proteolytic peptides that activate an immune response. Therefore, the detection of AMPH1 depletion in AD brains (De Jesus-Cortes et al., 2012) suggests that auto-AMPH1 antibodies could be a putative pathological biomarker.

Altogether, the results indicate that the reduction of AMPH1 protein and the detection auto-AMPH1 antibodies are a consequence or byproduct of tau-mediated neurodegeneration in the tauopathy mouse model JNPL3. The results also suggest that auto-immune responses could be an intrinsic component of tau-mediated neurodegeneration. Nevertheless, further experiments are needed to understand the molecular mechanisms that lead to the generation of auto-AMPH1 antibodies and its role in the pathobiology of tau-mediated neurodegeneration.

## Author contributions

Carlos J. Nogueras-Ortiz, Hector J. De Jesús-Cortes, and Jaime Vaquer-Alicea performed the experiments described in the manuscript. Carlos J. Nogueras-Ortiz and Irving E. Vega analyzed the data, interpreted the results, and wrote the manuscript.

### Conflict of interest statement

The authors declare that the research was conducted in the absence of any commercial or financial relationships that could be construed as a potential conflict of interest.

## Supplementary material

Dr. John F. Engelhardt kindly provided serum and spinal cord from three mSOD1 mice in a terminal study that used an unspecified drug. The mSOD1 mice showed severe motor impairment regardless of the drug treatment, indicating that the drug did not have an effect in the progression of neurodegeneration in this ALS mouse model. The results showed that AMPH1 is readily detected in terminally ill mSOD1 mice (Figure S1) and there is a weak detection of anti-AMPH1 antibodies after overexposure of the western blot membrane in only one of the terminally ill mSOD1 mice tested (Figure S2). Although these results suggest that in an ALS mouse model with motor impairment there is not reduction of AMPH1 protein and no detection of anti-AMPH1 antibodies in the serum, further experiments are required to validate these observations.

Figure S1 **AMPH1 protein level in the spinal cord of terminally-ill mSOD1 ALS model**. Western blot analysis was performed to detect AMPH1 and tau proteins in spinal cord protein extract from three different mSOD1 mice (e.g., ALS #1). Actin was used as loading control. The detected proteins are indicated at the right of the panel.

Figure S2 **No detection of anti-AMPH1 antibodies in terminally-ill mSOD1 ALS model**. The serum was obtained from the same mice used in Figure S1. The sera were used as primary antibody in a western blot assay where the antigen was recombinant GST-AMPH1. Two different exposures are shown to illustrate the different level of detection between the serum from JNPL3 mice and that from mSOD1 mice. Ponceau stained membrane are shown as loading control.

Table S1 **Description of mice used**. The table described the genotype, gender, age and motor impairment score of all mice used in the study. Mice were divided in three groups: (1) littermates—mice from the same progeny (brothers and sisters); (2) age-matched—mice that were paired based on their age at the time of euthanizing; (3) not matched—unpaired mice that were included in the respective age groups.
