# Supplementary figures and images for: Novel autoimmune response in a tauopathy mouse model
Source: Front Neurosci. 2014 Jan 10;7:277. doi: 10.3389/fnins.2013.00277 (PMC3887318; doi:10.3389/fnins.2013.00277)

Suppl. Figure 1. AMPH1 protein level in the spinal cord of terminally-ill mSOD1 ALS model

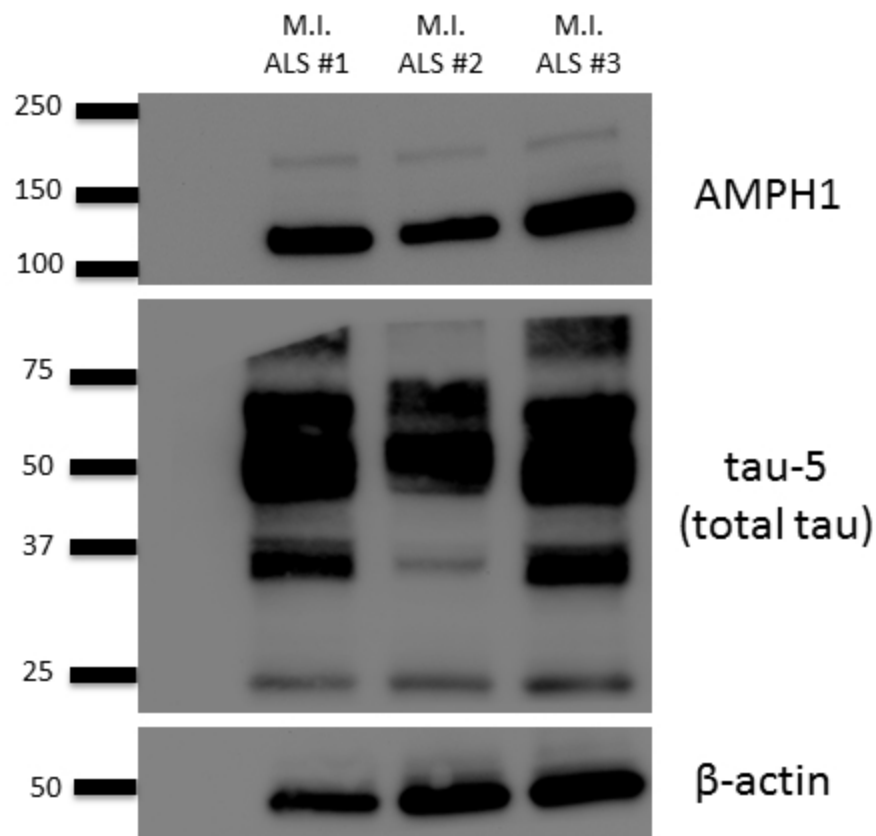

Supplement: Figure S1 — AMPH1 protein level in the spinal cord of terminally-ill mSOD1 ALS model. Western blot analysis was performed to detect AMPH1 and tau proteins in spinal cord protein extract from three different mSOD1 mice (e.g., ALS #1). Actin was used as loading control. The detected proteins are indicated at the right of the panel. [file Presentation1.PDF]

## Suppl. Figure 2. No detection of anti-AMPH1 antibodies in terminally-ill mSOD1 ALS model

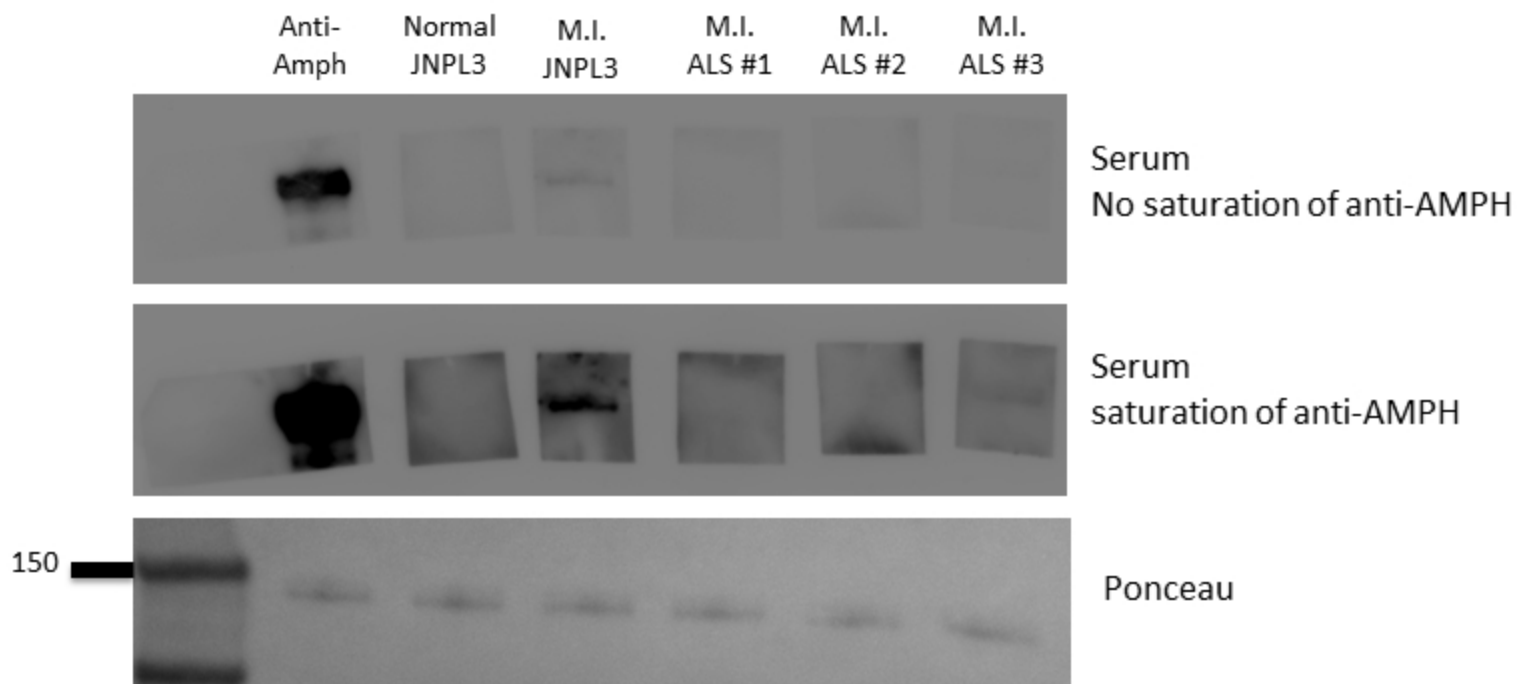

Supplement: Figure S2 — No detection of anti-AMPH1 antibodies in terminally-ill mSOD1 ALS model. The serum was obtained from the same mice used in Figure S1. The sera were used as primary antibody in a western blot assay where the antigen was recombinant GST-AMPH1. Two different exposures are shown to illustrate the different level of detection between the serum from JNPL3 mice and that from mSOD1 mice. Ponceau stained membrane are shown as loading control. [file Presentation2.PDF]
