# Supplementary material for: Novel autoimmune response in a tauopathy mouse model
Source: Front Neurosci. 2014 Jan 10;7:277. doi: 10.3389/fnins.2013.00277 (PMC3887318; doi:10.3389/fnins.2013.00277)
Supplement: Table S1 — Description of mice used. The table described the genotype, gender, age and motor impairment score of all mice used in the study. Mice were divided in three groups: (1) littermates—mice from the same progeny (brothers and sisters); (2) age-matched—mice that were paired based on their age at the time of euthanizing; (3) not matched—unpaired mice that were included in the respective age groups. [file Table1.PDF]

Table 1 | Mice utilized for experimentation.

Littermates

NTg	Sex	M.I.S.	JNPL3	Sex	M.I.S.	Age (M)
506	♂	0	513	♀	0	2.9
510	♀	0	515	♂	0	2.9
529	♀	0	533	♀	0	3.0
536	♂	0	535	♂	0	3.0
572	♀	0	566	♂	0	3.1
537	♂	0	540	♂	0	3.2
539	♂	0	538	♂	0	3.2
641	♀	0	639	♀	0	3.3
530	♀	0	531	♀	14	6.0
519	♂	0	518	♂	10	6.1
486	♂	0	488	♂	11	6.3
478	♂	0	480	♂	13	6.8
469	♂	0	466	♀	13	6.8
550	♀	3	551	♀	21	8.0
616	♂	0	621	♀	21	8.1
549	♂	2	552	♀	21	8.2
583	♀	0	581	♀	21	9.3
562	♀	5	561	♀	19	9.9
636	♀	13	637	♀	17	10.3
598	♂	10	599	♂	17	10.7
613	♀	0	611	♀	21	11.2
614	♀	0	612	♀	19	11.2
430	♀	15	428	♀	19	12.0
407	♂	0	411	♂	15	12.0
545	♂	10	546	♂	19	12.2
563	♂	13	565	♂	19	12.9
554	♂	15	555	♂	15	13.0
n = 27			n = 27			

Age matched

NTg	Sex	M.I.S.	JNPL3	Sex	M.I.S.	Age (M)
511	♀	0	522	♀	0	2.9
507	♂	0	498	♀	10	6.1
508	♂	0	499	♀	10	6.1
509	♂	0	503	♀	14	6.1
514	♀	0	516	♂	10	6.1
517	♂	0	512	♀	0	6.3
634	♀	0	623	♂	30	10.5
438	♂	0	439	♀	21	11.6
559	♀	10	560	♀	19	11.7
487	♂	10	475	♀	23	12.0
n = 10			n = 10			

Not matched

NTg	Sex	M.I.S.	Age (M)
644	♂	0	3.3
645	♂	0	3.3
**JNPL3**	**Sex**	**M.I.S.**	**Age (M)**
568	♂	0	3.1
470	♂	24	10.9
437	♂	10	12.0
n = 5			

M.I.S. – motor impairment score.
